# Report of the Diseases of Edinburgh, for April 1808

**Published:** 1808-06

**Authors:** John Roberton

**Affiliations:** Surgeon


### *Report of the Diseases of Edinburgh, for April* 1808; *by* Mr. JOHN ROBERTON, *Surgeon.*

THE weather about the beginning of this month was very variable, but upon the whole mild. The thermometer, though likewise variable, stood for the most part about 40. About the middle of the month we experienced cold, with sharp penetrating winds; and in the course of a few days, snow fell in an unusual quantity, considering the advanced period of the season. Vegetation, which, in the earlier part of the month, was making very rapid progress, suffered in consequence of this very much; and it was supposed, that the intense frost which immediately followed, would greatly injure the fruit blossoms. About the beginning of the last third of the month, snow fell in great quantities, accompanied by very cold winds. This sort of weather was, at the same period, very general all over this part of the island. Upon the hills immediately in the neighbourhood of Edinburgh, I was informed by some of the shepherds that, in consequence of this weather, it was customary for them to search every day for dead lambs, at which time they picked up great numbers of them. I have likewise been informed, that in the hilly countries to the northward of us, the lambs have suffered to a very great extent. In short, I believe such an untoward season has seldom happened with us during the remembrance of the oldest person now living. The trees in our neighbourhood exhibited a most strange appearance, for the leaves and some of the blossoms had advanced considerably, and on the same branches hung snow. The whole field beneath them likewise was one continued sheet of snow, and the ice on the lakes, particularly in the mornings, was of considerable thickness. During this period, as might have been expected, the thermometer was low. Near the end of the month a gradual thaw came on, and the face of Nature resumed the appearance of spring; still, however, the weather was by no means pleasant.—

The

The barometer, during most part of the month, continued rather low.

Inflammatory complaints, of various kinds, have, with few intermissions, (as may be seen in my previous Reports) been the prevailing complaints of this part of the world since the commencement of last winter; during which period, they have appeared in almost every variety of form, and in every degree of severity, from the most simple derangement of parts in consequence of inflammatory action, to fatal terminations in the course of a few hours illness. The secession of one might evidently have been traced to the formation of another; and, by accurate observation, some of them seem to have been so closely linked with each other, that they might rather have been taken for varieties of the same disease, than diseases specifically different.

Some cases of measles are still to be met with, and these certainly of the worst kind. The longer this disease seems to be protracted, its attacks appear to be proportionally severe. A great number, consequently, of these cases terminate fatally. From the length of time which this disease has, on the present occasion, prevailed, people in general pay little attention to its ravages; but the medical attendant, whose duty it is to explore the habitations of the diseased and wretched of every description, can mark the progress and decline of complaints, which, from their frequency, have long become so familiar to the public eye that their greatest state of malignity eludes general observation.

Soon after the desquamation of the eruption, in such cases as have lately come under my care, there has supervened a weak but quick pulse, a speckled or marbled appearance over the body, and dropsy, in one or other form, has in general terminated the patient's existence.

For the most part, I believe, the origin of epidemics, of almost every sort, at least as they appear in towns or cities, may be traced to have first made their appearance among the miserably constructed habitations of the poor, where nastiness of every description, and putridity in its most loathsome forms, are to be found. Epidemics of some kind or other, particularly in large and crowded cities, are almost constant. In a former Report I mentioned, that I had observed an epidemic fever attended, in its latter stages, by a petechial eruption, among these lower orders of society, which proved highly destructive to such as were affected by it. This fever, &c. does not seem to be be wholly confined to those people with whom it had its origin, as I have seen several cases of it in families of a very different description. I hope, however, by superior cleanliness and the well aired habitations of such families, it will be prevented from advancing farther. Attention to these circumstances during the existence of such fevers, which are evidently caused by an opposite state, appears to me a principal part of their treatment. Indeed, in many instances, I have, by these means alone, cured several slight cases of this disease. In the latter stages of it, when the petechiæ have made their appearance, and the pulse has sunk, (as almost always happens) I have recourse to the bark and wine, with the application of blisters, if necessary, to the head or breast; and by this treatment, assiduously persevered in, I have succeed ed in the removal of several cases which seemed of a very hopeless nature.

A very few cases of ophthalmia were to be met with in Edinburgh about the end of last month, but they were so trifling in their nature, as to excite no very particular attention. The disease still continued to exist with considerable violence among the soldiers of the Edinburghshire Militia, stationed in our neighbourhood.—About the beginning of the present month, however, it became more common, and before the end of the first third of the month, it had extended all over the city; and every description of people, young, middle-aged, and old, seemed equally affected by it.

Its approach was remarkably rapid, many of those affected by it being perfectly well on going to bed, but on rising next morning, found the whole balls of both their eyes covered by one uniform redness resembling a piece of scarlet cloth. Others felt as if there had suddenly been blown into their eyes a quantity of dust, or rather rough sandy particles, which they endeavoured to wipe away; but a very few minutes convinced them of their mistake, on observing their eyes affected as above described.

It would appear that the use of stimulating liquors, particularly ardent spirits, (too much used in this part of the world) had considerable influence in inducing this disease. A very great proportion of my patients put the question to me, if the use of a rather larger than usual quantity of spirits could produce it, as, they observed, that the affection commenced almost immediately after some excess of that kind; although many, I believe, were seized by it who indulged in no such practices.

Although

Although this disease was quick in its approach, and violent while it lasted, yet, in the majority of the cases that came under my observation, its duration was but very short, unless from the neglect of the patient or from his irregularity in living, when it was often protracted several days or even weeks. In general, however, it seldom existed beyond forty, or from that to forty-eight hours, and I have not observed that the pulse was remarkably affected during its continuance.

I may mention, that I paid very particular notice to its duration and effects in scrofulous constitutions; as, so far as my observation goes, in almost every disease with which such persons are affected, they are more roughly handled by them than those who are of a different habit. Consistently with this observation, the present ophthalmia was both more severe than common, and affected the parts more extensively in them than in others. In such instances, I have observed the inflammation not only affect the whole surface of the eye, but proceed considerably down the cheek.

In slight cases of this disease, it was easily and speedily removed by wearing a green shade over the eyes during the day; and on going to bed, taking a saline cathartic, and applying a piece of loaf bread dipped in cold water over the affected eyes, if (as was very commonly the case) they happened to be both affected.

In more severe cases, instead of cold water, bread dipped in a solution of acet. plumbi, or in distilled vinegar, with, at the same time, the administration of the cathartic, usually produced a cure in a few hours. In some instances, after the severity of the inflammation had abated, there remained blood-vessels of a considerable size running over the ball of the eye, which I divided. I tried a variety of ointments and washes, but without deriving any material benefit from their use, till I adopted this plan.

I was much disappointed in administering the same applications to patients of scrofulous habits, (the dividing of the vessels on the eye excepted, which I found of great benefit,) for instead of alleviating the inflammation, I in almost every case, observed that they rather tended to increase it, at least in extent if not in severity. The method I adopted in the cure of such persons, therefore, was the application of substances of a stimulating nature to the part affected, such as that of an ointment containing the red recipitate of mercury; washing the eyes with cold ardent ardent spirits, (which sometimes I found necessary to warm, when it frequently produced much better effects than when applied cold;) dropping into the eye, while some person held it open, several drops of tinct. thebaic; or blowing a quantity of this liquid through a quill into the affected eye. This sort of practice I have, on the present occasion, found preferable to any other with which I am acquainted.

*No. 4, St. James's Street.*

